# The critical role of humidity in modeling summer electricity demand across the United States

**DOI:** 10.1038/s41467-020-15393-8

**Published:** 2020-04-03

**Authors:** Debora Maia-Silva, Rohini Kumar, Roshanak Nateghi

**Affiliations:** 1 Environmental and Ecological Engineering, Purdue University, West Lafayette, IN 47906 USA; 2 Department Computational Hydrosystems, Helmholtz Centre for Environmental Research-UFZ, Leipzig, 04318 Germany; 3 School of Industrial Engineering, Purdue University, West Lafayette, IN 47906 USA

**Keywords:** Climate sciences, Climate change, Energy science and technology, Energy modelling

## Abstract

Cooling demand is projected to increase under climate change. However, most of the existing projections are based on rising air temperatures alone, ignoring that rising temperatures are associated with increased humidity; a lethal combination that could significantly increase morbidity and mortality rates during extreme heat events. We bridge this gap by identifying the key measures of heat stress, considering both air temperature and near-surface humidity, in characterizing the climate sensitivity of electricity demand at a national scale. Here we show that in many of the high energy consuming states, such as California and Texas, projections based on air temperature alone underestimates cooling demand by as much as 10–15% under both present and future climate scenarios. Our results establish that air temperature is a necessary but not sufficient variable for adequately characterizing the climate sensitivity of cooling load, and that near-surface humidity plays an equally important role.

## Abstract (web summary)

Cooling demand project largely ignores the role of humidity. Here the authors show that in many of the high energy consuming states, projections based on air temperature alone underestimates cooling demand by as much as 10–15% under both present and future climate scenarios, due to the neglected role of humidity.

## Introduction

Accurate predictions of demand are a key challenge in electricity adequacy planning. Climate, technological, and socioeconomic factors are commonly used in predictive models of electricity demand^1,2^ to ensure reliable planning and operation in the electricity sector by adequately balancing supply and demand. However, more frequent and intense climate extremes such as sustained heat waves^3,4^ cause unanticipated changes in load^5^, challenging the reliability of electricity demand predictions. This poses a significant risk to the resilient operation of power systems^6^. Specifically, unanticipated higher demand for space conditioning and refrigeration during heat waves has led to rolling outages with serious socio-economic and public health consequences^7–12^. In light of the expected increase in frequency and intensity of climate extremes^3^, climate-induced outages are an increasing risk to the resilient operation of the electric power infrastructure^13–17^.

Quantifying the climate-sensitive portion of the electricity demand, referred to as the climate-demand nexus, hinges on effective characterizations of the key measures of heat stress. Heat stress measures refer to a combination of temperature and humidity that capture human discomfort levels and its ability to efficiently dissipate heat to avoid life-threatening conditions^9,10,18,19^. Heat stress is generally associated with high morbidity and mortality rates during heat waves. Despite the rich literature in electricity demand prediction^2,20–23^, little prior work has focused on exploring the climate sensitivity of demand with respect to different measures of heat beyond air temperature. While climate science research establishes air temperature as an incomplete measure of the surface heat content^9,10,18,24,25^, the majority of the existing research on climate-demand nexus use air temperature—or features derived from air temperature, such as cooling and heating degree days—as key predictors^2,26–28^.

We address this gap by leveraging advanced data analytics to characterize the climate sensitivity of residential electricity demand as a function of a range of heat stress measures beyond air temperature, including dew point temperature, discomfort index (DI), heat index (HI), humidex, wet bulb temperature, and wet bulb globe temperature (see Methods). These measures are specifically chosen because they have been established in the literature as most effective in capturing heat stress^29^. However, no consensus has yet been reached on which measures are most predictive of the climate-demand nexus. We isolate the climate sensitivity of residential energy demand (see Methods), which has been established as the most climate-sensitive sector^15,16,30–34^. Thus, our results primarily reflect changes in demand due to climate variability. We focus on summer months—that is, May, June, July, and August—as the majority of the US states peak during summer, and climate extremes such as heat waves occur in the summer^35^.

The central thesis in this paper is that air temperature alone underestimates the climate-sensitive portion of energy demand for cooling. To test this hypothesis, we first comprehensively assess the role of air temperature in capturing the climate sensitivity of summer electricity load at a US national scale; then, we identify the measures of heat stress that are most predictive of the climate-demand nexus and finally we quantify the likely underestimation of models based on air temperature alone under current and future climate scenarios.

We use monthly aggregated, state-level electricity consumption data across the contiguous United States, extracted from the Energy Information Administration (EIA) public reports over the years of 1990–2016 as well as population data from the US Census Bureau, and harmonized climate data from NCEP North American Regional Reanalysis (NARR)^36^. The electricity consumption data is carefully adjusted to remove trends associated with demographic and technological changes to isolate the climate effects on energy demand^37^ (see Methods).

To quantify the effectiveness of air temperature alone in explaining the climate sensitivity of residential sector energy load, we develop two sets of models at the state level: air-temperature-only models, which only use surface air temperature as a predictor, and selected-features models, where data-driven variable selection is performed to identify the key measures of heat stress considered in this study. Comparing the results of the air-temperature-only and selected-features models, we determine the influence of each measure of heat stress on the overall climate sensitivity of demand. The predictive models are developed using a state-of-the-art, stochastic, non-parametric Bayesian ensemble-of-trees algorithm^38^ (see Methods), which has been shown to outperform other climate-demand nexus models in terms of predictive accuracy^1,14,15,31,39^.

We conclude that the air-temperature-only models underestimate residential energy consumption, specially for future climate scenarios. With that, we show that near-surface humidity has an equally important role in electricity prediction as air temperature.

## Results

### Climate features identification

**Fig. 1:**
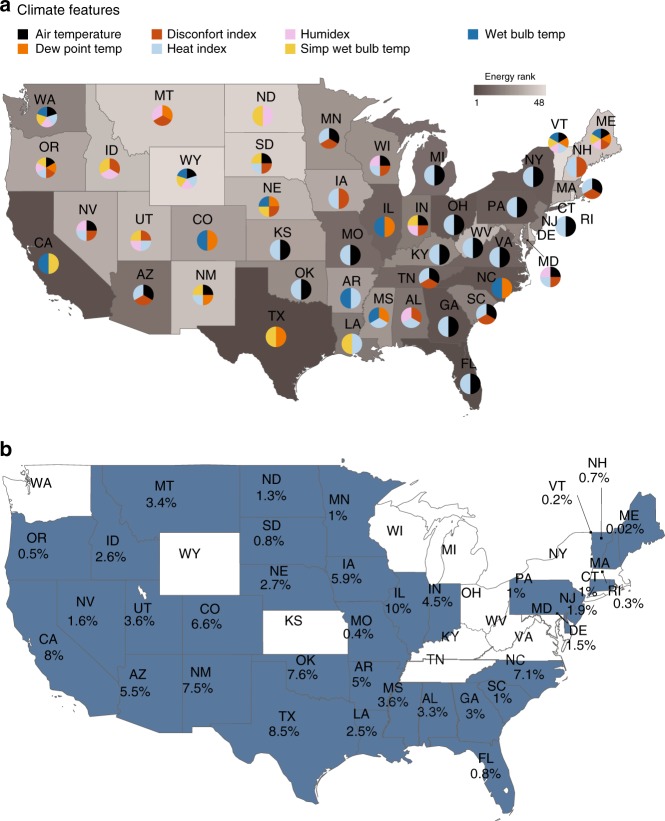
Key climate predictors of cooling demand. **a** States are shaded according to total energy consumption intensity (scale bar goes from 1 to 48, with darker tones representing higher intensity states in terms of energy consumption). The pie charts illustrate the state-level key measures of heat stress for predicting the climate-sensitive portion of electricity demand. The majority of states have up to two most important features. It is important to point out that climate sensitivity is not an exclusively geographical characteristic, and considering the population density weight method for the variables calculation (see Methods), it is possible to expect neighboring states to not necessarily have the same selected climate variables. Non-summer-peaking states with many selected features (e.g., Washington (WA) and Oregon (OR)) reveal relative climate insensitivity in comparison (Supplementary Note 1). Note that the top energy-consuming states of California (CA) and Texas (TX) do not include air temperature as the key predictors of the climate-sensitive portion of demand. **b** Highlighted states show percentage improvement in predictive accuracy when comparing the selected-features models to the air-temperature-only models (comparison based on out-of-sample RMSE). Source data are provided as a Source Data file.

Our results reveal substantial improvements in prediction accuracy in 35 states for the cooling demand as a function of both temperature and humidity over the air-temperature-only model. Fig. 1a illustrates the key predictors of the climate-sensitive portion of electricity demand across the 48 US states as identified by the selected-features model. A darker shade represents states with higher energy consumption and the pie charts depict the selected climate variables for each state. We find significant variability in the identified key measures of heat stress across the United States, illustrating the importance of conducting region-specific analysis in characterizing the climate-energy-demand nexus. The most energy-intensive states such as California and Texas, as well as many of the summer-peaking southern states (Supplementary Fig. 1 and Supplementary Note 1), benefit from the inclusion of measures of heat stress beyond air temperature—such as dew point temperature, wet bulb temperature, wet bulb globe temperature, and HI (see Methods)—in estimating the climate sensitivity of demand. This underscores the importance of accounting for near-surface humidity combined with temperature to better model human comfort levels and response to heat, which translates to residential cooling demand.

The highlighted states in Fig. 1b represent the extent in which the selected-features models outperformed air-temperature-only models in terms of predictive accuracy. Comparing the air-temperature-only models and selected-features models reveal that 35 states benefit from including measures of heat stress beyond air temperature, based on the comparison of root mean square error (RMSE) improvement in prediction accuracy (Fig. 1b). In addition to comparing model performances for the average consumption levels (i.e., 50th quantile predictions, Fig. 1b), we also compare the 90th quantile predictions (Supplementary Figs. 2 and 3). In this way, the effects of extreme heat events can be deduced from the higher quantiles of the aggregated electricity demand data. In other words, since heat waves typically coincide with the energy peaking summer months, the results for the 90th quantile RMSE improvement (Supplementary Fig. 3) reflect months with more intense and frequent heat events. We observe significant model improvements, in terms of out-of-sample RMSE, for a number of high-energy-consuming states of the order of 8% (7.7%) in California, 8.5% (21.1%) in Texas, 8.6% (26.1%) in Illinois, and 7.1% (9%) in North Carolina, for the 50th quantile predictions (and 90th quantiles predictions—Supplementary Fig. 3). The observed improvements are substantial. For instance, in a state such as Texas, the selected-features model’s average improvement of 8.5% in a given summer month such as August 2016 is equivalent to 1,498,968 MWh of energy consumption, which could sustain the residents of Austin-TX for more than 4 months^40–43^. In the same year and month, the 8% median increase in the state of California could sustain almost 1.5 million of Californians households^44^.

### Sensitivity analysis under future climate scenarios

To quantify the extent to which the air-temperature-only models underestimate the climate sensitivity of demand under different climate scenarios, we use projected climate data extracted from five widely used Global Circulation Models (GCMs).

The GCM-extracted climate data are used to train state-level air-temperature-only and selected-features models to predict for the reference period of 1991–2010, and the future period of 2031–2050 (Methods). Since the objective here is to evaluate the changes in demand pattern as a result of future climate change (i.e., to quantify the relative increase over the periods, as opposed to absolute values), reference period data from the GCM is used instead of the historical data to remove bias induced by the use of data from different sources in the sensitivity analysis. In other words, input data generated from the same source (i.e., the GCMs) over the two time periods of reference (1991–2010) and future (2031–2050) are used in the developed predictive framework to track the relative increase in the climate-sensitive portion of demand as estimated by the air-temperature-only versus selected-features models.

The 1991–2010 reference period was chosen to match the period of the observed data as closely as possible and, at the same time, the same length as the future period (i.e., 20 years). While the common practice in climate science research is making projections until 2100, that timeline is not appropriate in the energy sector, given the life span of the existing energy infrastructure^45–47^. The future period of 2031–2050 was chosen since energy infrastructure planning has the 2050 year as a comparable reference target in most energy projection and planning reports^48,49^.

**Fig. 2:**
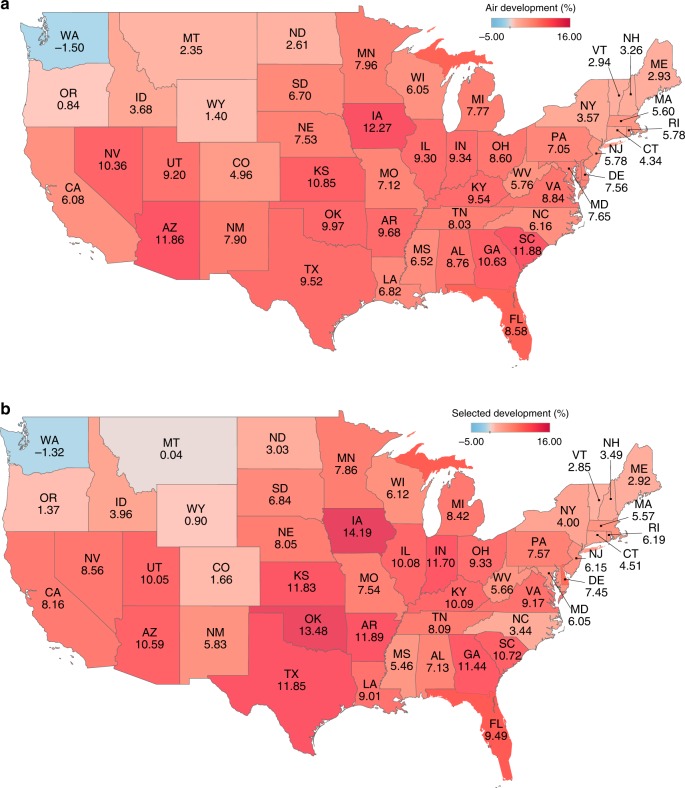
Projected increase in cooling demand. Increase in the climate-sensitive portion of the demand under climate change (scale bars go from − 5% to 16%) based on: **a** the air-temperature-only models and **b** the selected-features models. Note the significantly higher demand increases for the selected-features models (32 states showed higher values for the selected-features models), supporting the initial hypothesis that the air-temperature-based projections underestimate the climate sensitivity of demand. Source data are provided as a Source Data file.

The selected-features models project a significantly higher increase in the climate-sensitive portion of demand over time compared to the air-temperature-only models, when comparing the relative increase of each model (Fig. 2). The projections based on the selected-features models for the high-energy-consuming states of Texas, California, and Florida show a relative demand increase of about 12%, 8% and 10%, respectively, over the reference period (Fig. 2).

**Fig. 3:**
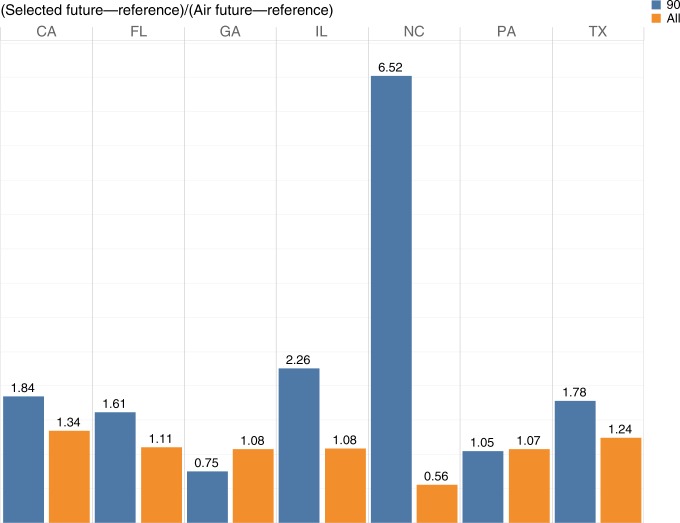
Relative increase in average and extreme projections. Seven out of the top ten energy-consuming states in the United States that showed improvement with selected-features model. The relative increase of projected demand by the selected-features model over the air-temperature-only model for the average consumption levels and 90th quantile predictions for the top energy-consuming states. Source data are provided as a Source Data file.

Figure 3 depicts the relative increase of projected demand by the selected-features model over the air-temperature-only model for the average consumption levels and 90th quantile predictions for the top seven energy-consuming states that presented an improvement in prediction accuracy in the historical period. Figure 3 enables the comparison between the ratios of the projections based on the selected features over the air-temperature models for 90th percentile as well as the average values, as shown in the equation below. Complete results for all states are shown in Supplementary Fig. 4.

1 $${\frac{{\mathrm{Selected}}\,\, {\mathrm{features}}\,\, {\mathrm{future}}_{{\,}2031{\hbox {-}}2050} - {\mathrm{Selected}}\,\, {\mathrm{features}}\,\, {\mathrm{reference}}_{{\,}1991{\hbox {-}}2010}}{{\mathrm{Air}} {\hbox {-}} {\mathrm{temperature}} {\hbox {-}} {\mathrm{only}} \,\,{\mathrm{future}}_{{\,}2031{\hbox{-}}2050} - {\mathrm{{Air}}} {\hbox {-}} {\mathrm{temperature}} {\hbox {-}} {\mathrm{{only}}}\,\, {\mathrm{{reference}}}_{{\,}1991{\hbox{-}}2010}}}$$

This comparison reveals that the underestimation in energy demand attributable to the air-temperature-only models is substantially more significant for the projections of higher demand values that are typically associated with periods of intense heat stress months—where the resilient operation of the grid is most challenged and unexpected demand spikes lead to supply inadequacy and thereby increase the risks of morbidity and mortality.

## Discussion

The existing projections of climate-induced demand increase based on rising air temperatures alone ignore the fact that rising temperatures are associated with increased humidity—a lethal combination that increase morbidity and mortality in the absence of adequate cooling capacity during extreme heat events. We show that air temperature is a necessary but not sufficient measure to characterize residential space cooling demand during summer times. Humidity levels are also critical in capturing what truly reflects heat sensation for the human body^9^. Ignoring the role of humidity leads to underestimating the climate sensitivity of demand, challenging the resilient operation of power systems—especially under future warming scenarios where summertime energy production will be further constrained^50^. Inadequate supply to meet rising demand will have significant socio-economic costs due to adverse health effects—particularly among the most vulnerable population—and warrants rapid and costly investments in energy infrastructure expansion as well as adaptation measures^26^.

We propose a data-driven framework to quantify the extent of underestimation of the climate-sensitive portion of cooling load, attributable to the use of air temperature alone as a measure of heat stress. Our results reveal a significant increase in predictive accuracy (of the order of 8% for high-energy-consuming states such as California and Texas) in characterizing the climate-sensitive portion of demand through a more holistic inclusion of measures of heat stress. The results based on projecting demand under future climate scenarios are consistent, in that models based on air temperature alone show a systematic underestimation of the climate-sensitive portion of demand. The underestimation is particularly pronounced at the upper tail of the demand distribution, suggesting that the projected increase would be almost twice as large when considering measures of heat stress beyond air temperature in high-energy states such as California, Florida, and Texas.

It is important to note that our results pertain to only the climate-sensitive portion of the residential cooling demand. To project changes in total residential cooling demand under future climate scenarios, additional information regarding economic and population growth as well as technology and demographic changes need to be considered. What we wish to highlight here is that, under similar cooling technology, the existing projections based on air temperature alone substantially underestimate the anticipated demand increase under future warming scenarios, which could lead to inadequate and ineffective investments in capacity expansion and demand response programs as well as adaptation measures. Furthermore, while this study is focused on the residential energy demand, the methodology can be extended to other climate-sensitive sectors^51^.

## Methods

### Observed electricity consumption data

Monthly electricity consumption data was extracted from the EIA^52^ over the years of 1990–2016 at a state level, with a total of 68 months per state (4 months per year for a total of 17 years). The source file includes electricity consumption data in Megawatt hours (converted to kWh), separated by sector: residential, commercial, industrial, transportation and others. The electricity consumption of the residential sector was the focus of the analysis presented in this paper. The residential electricity consumption data was detrended to isolate the climate effects on energy demand from techno-demographic changes such that increases in energy were not attributable to non-climatic factors such as technology shifts and population increase^16,37^. The residential energy consumption data in each state was first normalized using state-level population data—from the US Census Bureau^41^—to obtain a per-capita electricity demand, and then detrended based on the following steps:

2 $$E(y)=\frac{\mathop{\sum }\nolimits_{y=1}^{{n}_{\mathrm{years}}}\mathop{\sum }\nolimits_{m=1}^{12}E(m,y)}{{n}_{\mathrm{years}}},$$

where the total years, *n*_years_, range from 1990 to 2016, *m* denotes the month, and *y* the year.

Then, an adjustment factor was calculated for each year as the sum of the per-capita consumption per month divided by the yearly consumption *E*(*y*). This process was repeated for all states as follows:

3 $${F}_{\mathrm{adj}}=E{(y)}^{-1}\mathop{\sum }\limits_{m=1}^{12}E(m,y).$$

Finally, the adjusted energy consumption was calculated by dividing the monthly consumption by the adjustment factor:

4 $$E{(m,y)}_{\mathrm{adj}}=E(m,y)/{F}_{\mathrm{adj}}.$$

The detrended monthly electricity consumption data (described above) is referred to as observed electricity consumption data. This is a widely used method for trend-adjusting monthly aggregated regional data. For a comparative assessment of different de-trending methodologies please refer to Bessec and Fouquau^53^.

### Observed climate data

The observed climate data was acquired at a sub-daily time scale for the period of 1990–2016 from the NCEP NARR^36,54,55^, aggregated at a monthly level to match the temporal scale of electricity consumption data, and weighted by population so as to give a greater weight to areas with higher population density when aggregating to the state level. The input climate data include air temperature, dew point temperature, DI, HI, humidex, wet bulb temperature, and wet bulb globe temperature. All climate variables use a combination of temperature, humidity, and pressure.

Dew point refers to the temperature in which air is saturated with water vapor, calculated in this paper using the equation below:

5 $${t}_{\mathrm{d}}\approx t-\left(\frac{{100}-\mathrm{RH}}{{5}}\right),$$

where *t*_d_ is the dew point temperature and *t* is air temperature in Celsius, and RH stands for relative humidity in percent (same RH as in the other equations in this section)^56^.

Wet bulb temperature is the lowest temperature to which air can be cooled by water evaporation at a constant pressure. In this paper, it follows the equation:

6 $${T}_{\mathrm{W}}={T}_{\mathrm{W}}-\frac{f({T}_{\mathrm{W}};\pi )-{(C/{T}_{\mathrm{E}})}^{\lambda }}{f^{\prime} ({T}_{\mathrm{W}};\pi )},$$

where $\pi ={(p/{p}_{0})}^{1/\lambda }$ is the Exner function, used for scaling, *λ* is the inverse of the Poisson constant for dry air (3.504), *p* pressure (mb), *p*_0_ the reference pressure (1000 mb); *C* is the freezing temperature in Kelvin, and *T*_E_ the equivalent temperature, which is the moist potential temperature scaled by *λ*. For reviewing the complete derivation of the equation, refer to the Appendix A of refs. ^29,57^.

DI was developed in the 1950s to calibrate air conditioners and further adapted by the Israeli Defense Force as a main measure of heat stress:

7 $${\mathrm{DI}}={0.5}{T}_{\mathrm{W}}+{0.5}{T}_{\mathrm{C}},$$

where *T*_W_ stands for wet bulb temperature, RH is percentage relative humidity, and *T*_C_ is the temperature in Celsius. DI is unitless^29,58,59^.

Wet bulb globe temperature (sWBGT) is the heat stress measure in direct sunlight:

8 $${\mathrm{sWBGT}}={0.56}{T}_{\mathrm{C}}+\frac{{0.393}{e}_{\mathrm{RH}}}{{100}}+{3.94},$$

where *e*_RH_ = (RH/100)*e*_sPa_; wet bulb temperature used here is unitless and widely used.

*e*_RH_ represents the vapor pressure in pascals and is calculated from RH in percentage and saturated vapor pressure^29,60^.

Humidex was developed for the Meteorological Service of Canada. It is a unitless^29,61^ index, aiming to explain what the temperature feels like for the human body:

9 $${\mathrm{Humidex}}={T}_{\mathrm{C}}+\frac{{5}}{{9}}\left(\frac{{e}_{\mathrm{RH}}}{{100}}-{10}\right).$$

HI is also called apparent temperature, describing what the temperature feels like to the human body when relative humidity is combined with air temperature:

10 $${\mathrm{HI}}=	-{42.379}+{2.04901523}{T}_{\mathrm{F}}+{10.14333127}{\mathrm{RH}}-{0.22475541}{T}_{\mathrm{F}}{\mathrm{RH}}\\ 	-\,{6.83783}x1{0}^{-3}{T}_{\mathrm{F}}^{2}-{5.481717}x1{0}^{-2}{\mathrm{RH}}^{2}+{1.22874}x1{0}^{-3}{T}_{\mathrm{F}}^{2}{\mathrm{RH}}\\ 	+\,{8.5282}x1{0}^{-4}{T}_{\mathrm{F}}{\mathrm{RH}}^{2}-{1.99}x1{0}^{-6}{T}_{\mathrm{F}}^{2}{\mathrm{RH}},$$

where (*T*_F_) denotes the air temperature, and HI are measured in degrees Fahrenheit^29,62^.

### Projected climate data

Five different GCMs were used in this paper, namely, Geophysical Fluid Dynamics Laboratory Earth Systems Model (GFDL-ESM2M), Hadley Global Environment Model 2-Earth System (HadGEM2-ES), IPSL Earth System Model for the 5th IPCC report (IPSL-CM5A-LR)^63^, Atmospheric Chemistry Coupled version of MIROC-ESM, a Earth System model (MIROC-ESM-CHEM), and the Norwegian Earth System Model (NorESM1-M). The data are considered under the emission scenario that has an end-of-century radiative forcing equal to 8.5 W m^−2^, a Representative Concentration Pathway that is characterized by high greenhouse emission levels (RCP8.5)^15,64^. This global data is approximated by a 0.5° spatial resolution (~50 km)^65^ and is processed from 1950 to 2099. The same variables were calculated for the projected data, namely, dew point temperature, DI, HI, humidex, wet bulb temperature, and wet bulb globe temperature. Two periods were extracted from the projected data, namely, the reference period of 1991–2010, and the future period of 2031–2050.

There were two distinct stages in model development and analysis, namely, predictive model development using the observed data and sensitivity analysis using the projected data (Supplementary Fig. 5). In the predictive model development stage, electricity consumption data, climate data, and population data were aggregated and normalized at the state level, as explained earlier, yielding monthly observed data for 48 states. Using these data sets, the development of air-temperature-only models and selected-features models involved three steps: for the selected-features model, variable selection was performed to identify the key predictors of cooling demand (details in the next section: BART (Bayesian Additive Regression Trees) algorithm and modeling process); then, the model hyper-parameters were tuned (through cross-validation), and finally a ten-fold cross-validation was performed to assess the predictive accuracy of the final models. For the air-temperature-only models, the same process was followed with the exception of the variable selection step, as there was only one independent variable. After developing these statistical models, the results were processed with the collection of in-sample (training set, 90%) and out-of-sample (test set, 10%) *R*^2^ and RMSE values. The comparative assessments between the models were conducted based on the out-of-sample values of errors (RMSE results shown on Supplementary Figs. 2 and 3 and *R*^2^ results shown on Supplementary Figs. 6 and 7).

In the sensitivity analysis stage, projected climate data from the five GCMs over the reference period (1991–2010) and future period (2031–2050) were used as inputs to the previously developed predictive models. This generated four sets of data, namely, air temperature, reference: estimates from the air-temperature-only model using the reference period data; air temperature, future: estimates from the air-temperature-only model using the future period; selected features, reference: estimates from the selected-features model for the reference period; and finally, the selected features, future, estimates from the selected-features model for the future period. This allowed for calculating the delta change over time between the same models (i.e., calculating the difference between selected-features models over the periods of 2031–2050 and selected 1991–2010). Similar analysis was also conducted on the 90th quantile of data, as electricity inadequacy during extreme heat episodes is typically associated with the tail of the demand distribution.

### BART algorithm and modeling process

We leveraged a non-parametric Bayesian ensemble-of-trees algorithm to characterize the climate sensitivity of electricity consumption, since the algorithm was shown to outperform other climate-demand nexus models in terms of predictive accuracy^1,14,15,31,38,39^. The independent variables in the development of the BART models were heat stress measures, and the response variable was state-level electricity consumption. Models were trained and tested using a 10-fold cross-validation technique^66^.

The non-parametric Bayesian sum-of-trees model can be represented using the equation below^38,67^:

11 $${Y}=\mathop{\sum }\limits_{j=1}^{m}g(x;{T}_{j},{M}_{j})+\varepsilon ,\varepsilon \sim N(0,{\sigma }^{2}).$$

Here, *Y* denotes the response variable, *g* denotes the regression tree function, *T*_*j*_ denotes individual tree structures, *M*_*j*_ denotes a set of parameters (e.g., mean values) associated with the tree nodes. BART is the sum of *m* total trees. The decision rules associated with each tree involve binary splitting of the predictor space. So for a given *T* and *M*, *g*(*x*; *T*, *M*) represents a tree with parameters *M* assigning the binary splits for the terminal nodes of *T*. *ε* is the stochastic irreducible error, assumed to be normally distributed with a constant variance^38^.

The BART algorithm uses a prior over the sum-of-trees parameters to regularize the trees, that is, to weaken the effect of individual trees with the goal for better prediction when adding all decision trees. A prior is imposed on all parameters, (*T*_*j*_, *M*_*j*_) [∀*j* = 1, . . . , *m*] and *σ*. This regularization is made to keep individual trees effects from being unduly influential over the entire sum of trees. Without a regularization, large tree components would overwhelm the sum of trees and would diminish the benefits of leveraging additive tree structures. The prior regularization can be mathematically represented as:

12 $${\rm{p}}({M}_{j}| {T}_{j})=\mathop{\prod }\limits_{i}^{m}p({\mu }_{ij}| {T}_{j}),\quad{\mathrm{where}}\; {\mu }_{ij}\in {M}_{j},$$

where tree components *T*_*j*_ and *M*_*j*_ are assumed to be independent of each other and of *σ*.

As mentioned earlier, to assess the role of air temperature in capturing the climate sensitivity of electricity consumption compared to other measures of heat stress, two models were developed for each state: the air-temperature-only model, and the selected-features model. Air-temperature-only models were constructed with a single input variable of surface temperature and electric demand as the response variable. To develop the selected features a data-driven variable selection algorithm was harnessed. Variable selection: specifically, the variable selection algorithm involved identifying the variables most frequently used as decision/splitting rules in the sum-of-tree model^38^. The calculation was then averaged over 100 model iterations to achieve stable estimates throughout the sum-of-trees distribution. This is a reasonably robust approach for variable selection using the Bayesian ensemble-of-trees algorithm^38,67^.

### Pseudo-code

Feature selection is performed for 48 states before running BART and out-of-sample RMSE is averaged over 20 models (cross validation performed at line 3). Code was performed in parallel cores.

### Algorithm 1

BART Machine Model Fit

 1: **for** *i* ∈ {1 : 48} **do**

 2:      **for** *k* ∈ {1 : 20} **do**

 3:         $$BartMachin{e}_{k},RMS{E}_{k},{R}_{k}^{2}$$

 4:     **end** **for**

 5:     *RMSE*_*i*_ = *mean*(*RMSE*_*k*_) ~ *k* ∈ 1 : 20

 6: $${R}_{i}^{2}=mean({R}_{k}^{2}) \sim k\in 1:20$$

 7: **end** **for**

### Reporting summary

Further information on research design is available in the Nature Research Reporting Summary linked to this article.

## Supplementary information

Supplementary Information

Reporting Summary

## Data Availability

All data sets used in this study are publicly available from the referenced sources.

## Source data

Source Data

## Code availability

Code is available at the corresponding author’s Github along with pre-processed data [https://github.com/DebsM/humidity_paper_codes].
