# Supplementary material for: The critical role of humidity in modeling summer electricity demand across the United States
Source: Nat Commun. 2020 Apr 3;11:1686. doi: 10.1038/s41467-020-15393-8 (PMC7125155; doi:10.1038/s41467-020-15393-8)
Supplement: Supplementary file 1 — Supplementary Information [file 41467_2020_15393_MOESM1_ESM.pdf]

The critical role of humidity in modeling summer electricity demand across the United States

Authors: Maia-Silva et al.

The critical role of humidity in modeling summer electricity demand across the United States

Debora Maia-Silva *[1], Rohini Kumar †[2], and Roshanak Nateghi[1,3]

[1]Environmental and Ecological Engineering, Purdue University, West Lafayette, IN, 47906
[2]Department Computational Hydrosystems, Helmholtz Centre for Environmental Research-UFZ, Leipzig, Germany, 04318
[3]School of Industrial Engineering, Purdue University, West Lafayette, IN, 47906

*Corresponding Author; E-mail address: dmaiasil@purdue.edu
†Corresponding Author; E-mail address: rohini.kumar@ufz.de

Supplementary Information

Supplementary Figures

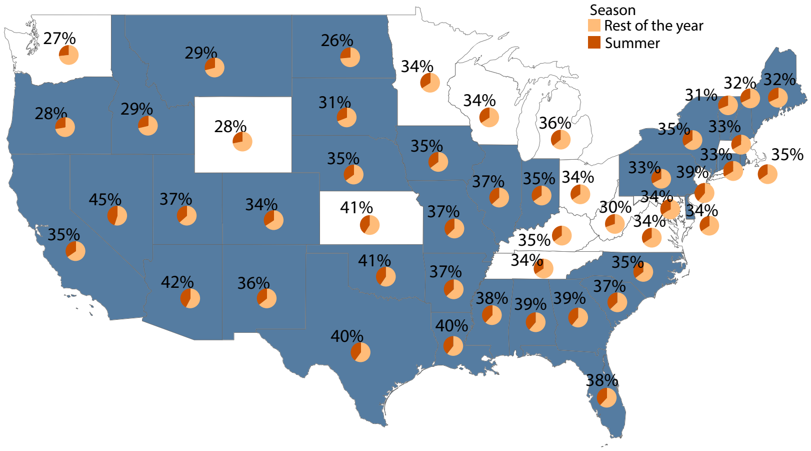

Supplementary Figure 1: Highlighted states represent areas where the selected-features model outperform the air-temperature-only models in terms of predictive accuracy.

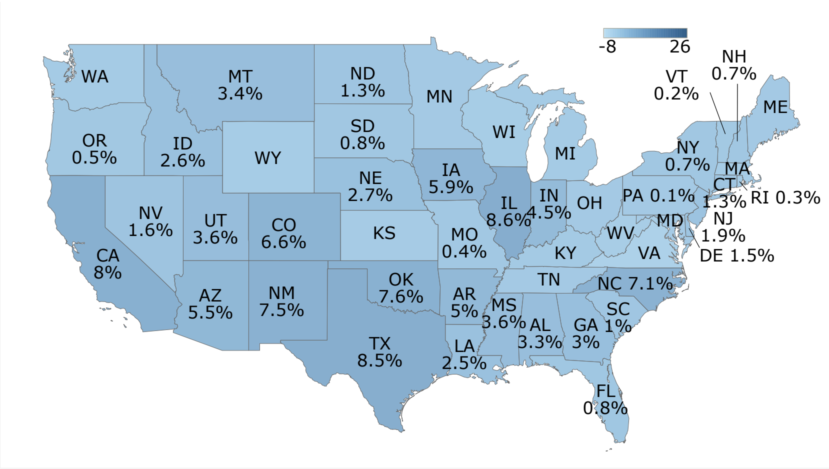

(a)

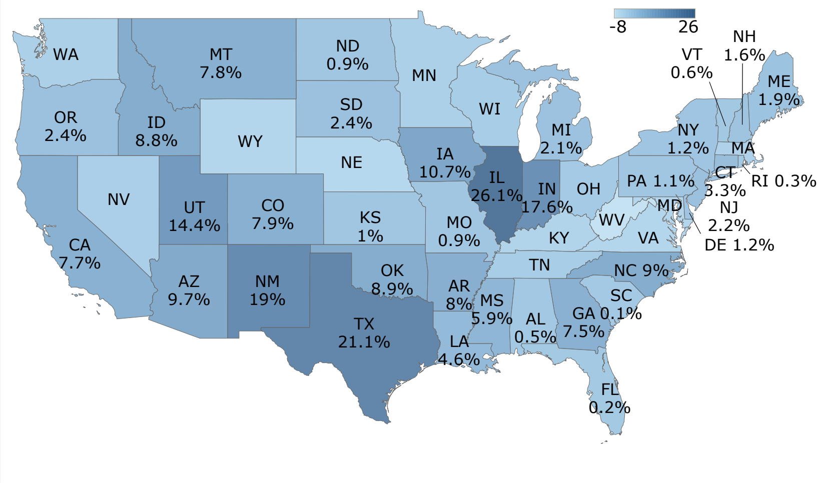

(b)

Supplementary Figure 2 and 3: Scale bars go from -8 to 26%. The relative improvement of the selected-features models over air-temperature-only models in terms of predictive accuracy—based on out-of-sample RMSE values—for the 50th quantile (a), and 90th quantile predictions (b). The RMSE values have not been included in areas where the state-level selected-features models did not out-perform the air-temperature-only models.

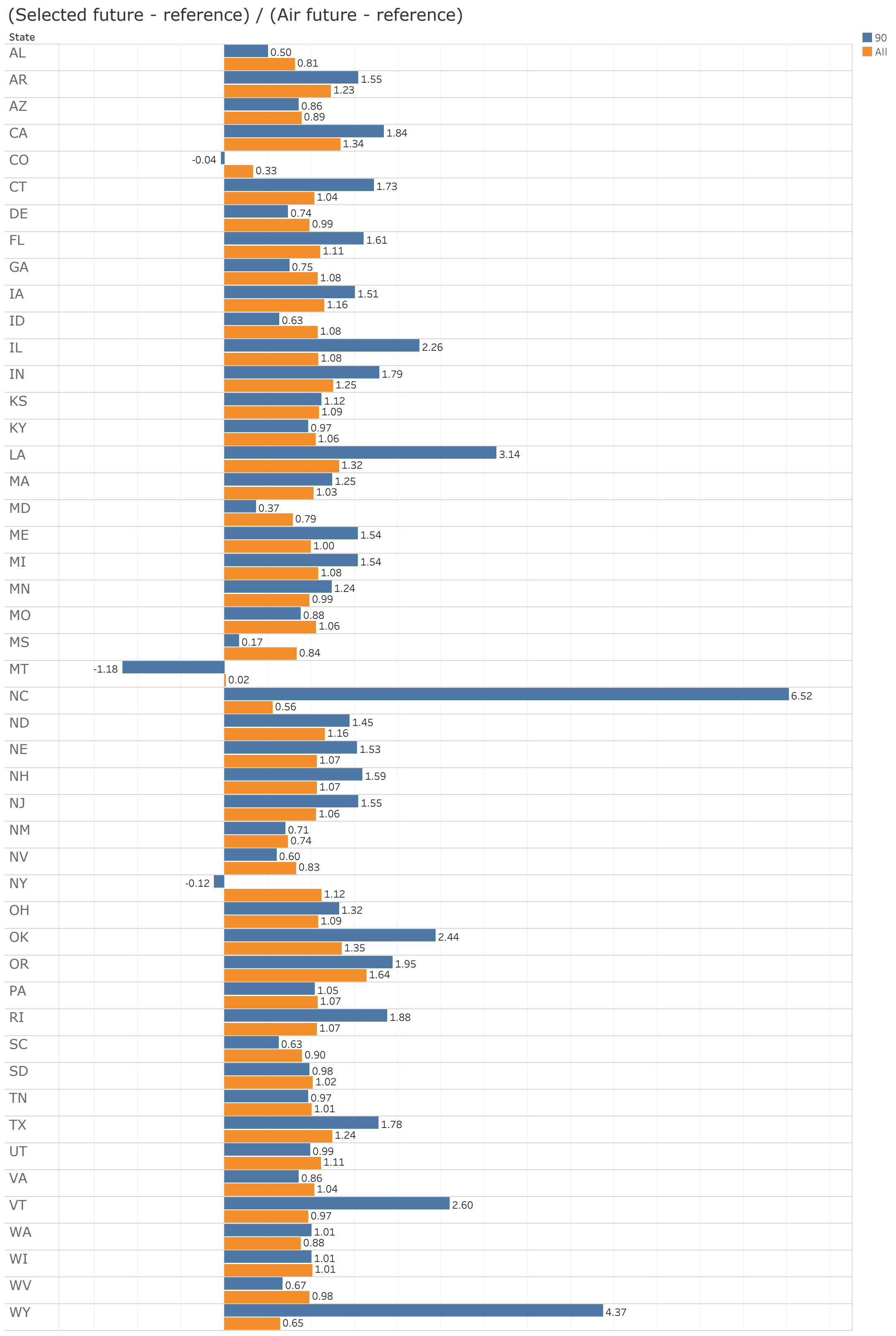

Supplementary Figure 4 The relative increase of projected demand by the selected-features model over the air-temperature-only model for average consumption levels and 90th quantile predictions for all states.

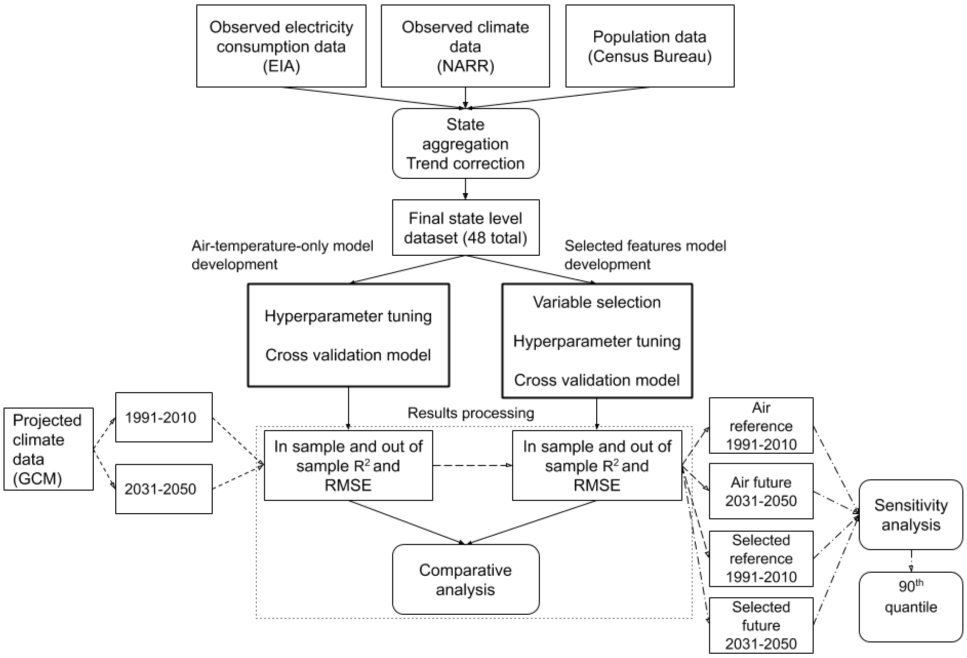

Supplementary Figure 5: Flow chart with the implemented methodology, complementing Methods section Projected climate data. The two stages in model development (modeling with observed data and sensitivity analysis with projected data) are represented by the vertical direction of the flow chart (from the observed data collection until results processing, continuous arrows) and the horizontal direction of the flow chart (from projected climate data until the 90th quantile sensitivity analysis, dashed arrows).

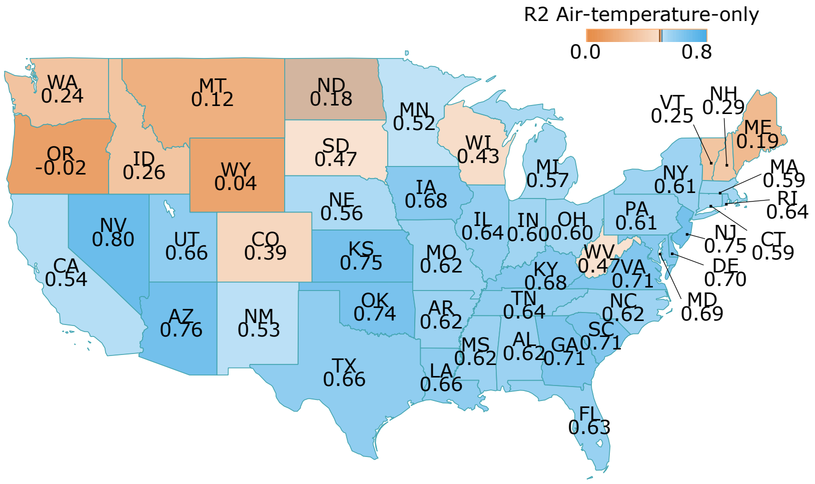

(a)

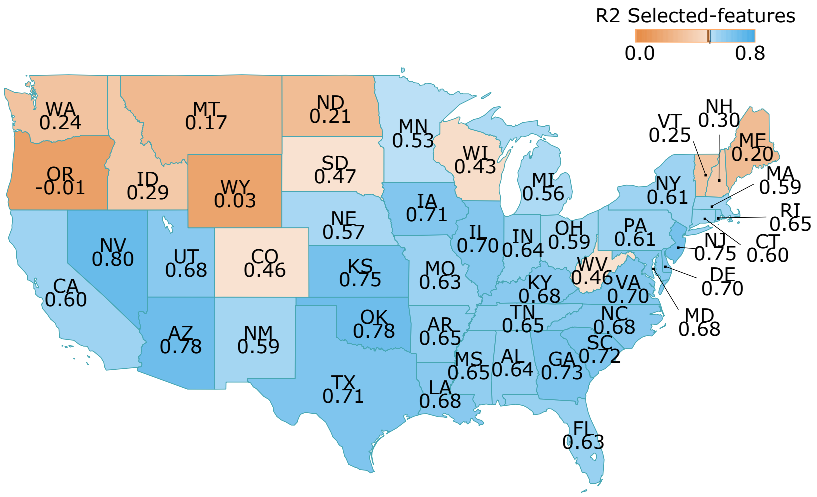

(b)

Supplementary Figure 6 and 7: (a) Out of sample R^2 air-temperature-only models. Red tones represent values negative or close to zero. Values in green are greater than 0.5; same color scheme for both R^2 plots.(b) Out of sample R^2 Selected features. As in the air models, the northwest region did not present a good fit and the southern region presents good fit with clear improvements, like in TX, from 0.66 to 0.71.

Supplementary Note 1

Energy consumption during summer represents a high percentage of total consumption in the U.S. The observed data from 1990 to 2016 reveal that the summer months (i.e., May, June, July and August) account for an average of 35% of total energy consumption, and even exceeding 40% in some states (Supplementary Figure 1). States which consumption is equal greater than 40% represent a highly meaningful peak.

The highlighted states in Supplementary Figure 1 signify areas where accounting for humidity in addition to surface temperature leads to an improved accuracy in characterizing the climate sensitivity of electricity demand. In other words, the selected-features models outperform the air-temperature-only model, in terms of predictive accuracy, in the highlighted states.
