# Supplementary material for: The critical role of humidity in modeling summer electricity demand across the United States
Source: Nat Commun. 2020 Apr 3;11:1686. doi: 10.1038/s41467-020-15393-8 (PMC7125155; doi:10.1038/s41467-020-15393-8)
Supplement: Supplementary file 3 — Source Data [file 41467_2020_15393_MOESM3_ESM.zip › Source Data/figures.zip!/si_figure_8.pdf]

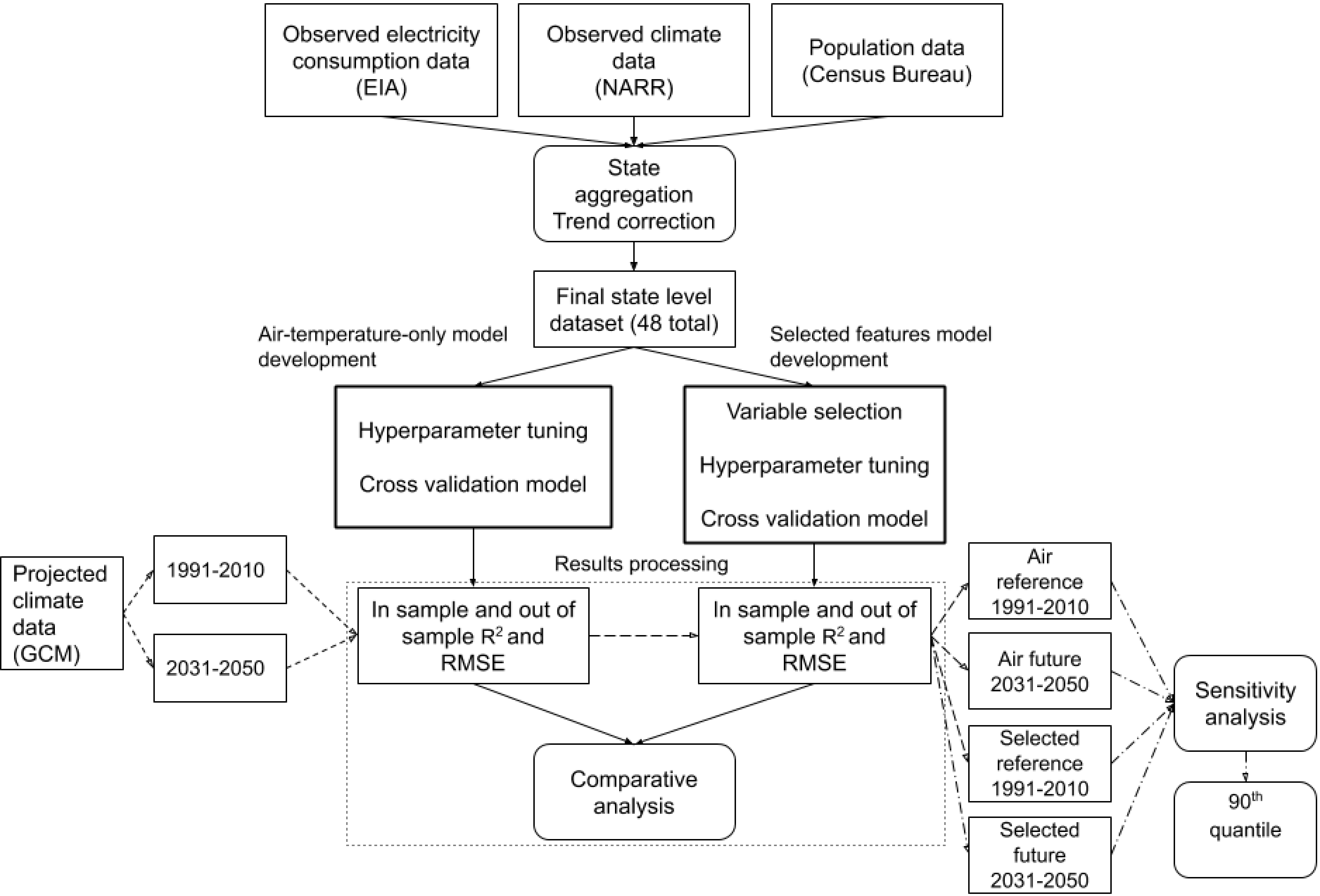

Observed electricity consumption data (EIA)
Observed climate data (NARR)
Population data (Census Bureau)
State aggregation Trend correction
Final state level dataset (48 total)
Air-temperature-only model development
Selected features model development
Hyperparameter tuning
Cross validation model
Variable selection
Hyperparameter tuning
Cross validation model
Projected climate data (GCM)
1991-2010
2031-2050
Results processing
In sample and out of sample R2 and RMSE
In sample and out of sample R2 and RMSE
Comparative analysis
Air reference 1991-2010
Air future 2031-2050
Selected reference 1991-2010
Selected future 2031-2050
Sensitivity analysis
90th quantile
